# Total Synthesis, Cytotoxic Effects of Damnacanthal, Nordamnacanthal and Related Anthraquinone Analogues

**DOI:** 10.3390/molecules180810042

**Published:** 2013-08-20

**Authors:** Muhammad Nadeem Akhtar, Seema Zareen, Swee Keong Yeap, Wan Yong Ho, Kong Mun Lo, Aurangzeb Hasan, Noorjahan Banu Alitheen

**Affiliations:** 1 Faculty of Industrial Sciences & Technology, Universiti Malaysia Pahang, Lebuhraya Tun Razak 26300, Kuantan Pahang, Malaysia; E-Mail: seema_zareen@yahoo.com; 2 Institute of Bioscience, Universiti Putra Malaysia, UPM Serdang 43400, Selangor Darul Ehsan, Malaysia; E-Mail: skyeap2005@yahoo.com; 3 School of Biomedical Sciences, The University of Nottingham Malaysia Campus, Jalan Broga, Semenyih 43500, Selangor, Malaysia; E-Mail: wanyongho@gmail.com (W.Y.H.); 4 Department of Chemistry, University of Malaya, Kuala Lumpur 50603, Malaysia; E-Mails: kimlo@um.edu.my (K.M.L.); flavonoids@hotmail.com (A.H.); 5 Faculty of Biotechnology and Bimolecular Sciences, Universiti Putra Malaysia, UPM Serdang 43400, Selangor Darul Ehsan, Malaysia; E-Mail: noorjahan@biotech.upm.edu.my

**Keywords:** Anthraquinone, damnacanthal, nordamnacanthal, MCF-7, K-562, cytotoxic effects

## Abstract

Naturally occurring anthraquinones, damnacanthal (**1**) and nordamnacanthal (**2**) were synthesized with modified reaction steps and investigated for their cytotoxicity against the MCF-7 and K-562 cancer cell lines, respectively. Intermediate analogues 2-bromomethyl-1,3-dimethoxyanthraquinone (**5**, IC_50_ = 5.70 ± 0.21 and 8.50 ± 1.18 μg/mL), 2-hydroxymethyl-1,3-dimethoxyanthraquinone (**6**, IC_50_ = 12.10 ± 0.14 and 14.00 ± 2.13), 2-formyl-1,3-dimethoxyantharquinone (**7**, IC_50_ = 13.10 ± 1.02 and 14.80 ± 0.74), 1,3-dimethoxy-2-methylanthraquinone (**4**, IC_50_ = 9.40 ± 3.51 and 28.40 ± 2.33), and 1,3-dihydroxy-2-methylanthraquinone (**3**, IC_50_ = 25.60 ± 0.42 and 28.40 ± 0.79) also exhibited moderate cytotoxicity against MCF-7 and K-562 cancer cell lines, respectively. Other structurally related compounds like 1,3-dihydroxyanthraquinone (**13a**, IC_50_ = 19.70 ± 0.35 and 14.50 ± 1.28), 1,3-dimethoxyanthraquinone (**13b**, IC_50_ = 6.50 ± 0.66 and 5.90 ± 0.95) were also showed good cytotoxicity. The target compound damnacanthal (**1**) was found to be the most cytotoxic against the MCF-7 and K-562 cancer cell lines, with IC_50_ values of 3.80 ± 0.57 and 5.50 ± 1.26, respectively. The structures of all compounds were elucidated with the help of detailed spectroscopic techniques.

## 1. Introduction

Anthraquinone compounds, especially anthracyclines, have long been used as effective anticancer drugs. Depending on their chemical structure, anthraquinone drugs can kill tumor cells by diverse mechanisms, involving different initial intracellular targets that normally contribute to drug-induced toxicity [1,2,3]. Anthraquinones are known as “multipotent antioxidants”, as they are molecules that besides antioxidant activity possess additional pharmacological activities such as inhibition of platelet-aggregation or display antineoplastic and anticancer activities [4,5]. Many anthraquinones also display various biological activities such as antimicrobial, antifungal, hypotensive, analgesic, antimalarial [6,7,8,9,10,11], antileukemic, mutagenicity and anti-inflammatory properties [12,13,14]. Natural anthraquinones from *Damnacanthus subspinosus* and *Morinda parvifolia* have long been used in traditional medicine for the treatment of cancer [15]. The discovery of new compounds with antitumor activity has become one of the most important challenges in medicinal chemistry. The detail study on anthraquinones has revealed that a range of DNA-recognizing molecules that act as antitumor agents, including groove binders, alkylating and intercalator compounds. DNA intercalators have attracted particular attention because of their antitumor activity. For example, a number of acridine and anthracycline compounds are excellent DNA intercalators that are now on the market as chemotherapeutic agents [15,16]. Substituted anthraquinones such as rubiadin, subspinosin and morindaparvin are widely distributed in nature and are known to display various pharmacological activities [17,18,19,20,21]. Previously, we have reported the antitumor and anti-oxidant activities of anthraquinones isolated from *Morinda elliptica* [22].Recently, we have also reported the cytotoxic and immunomodulatory effects of damnacanthal and nordamnacanthal against different cell lines [23,24]. Damnacanthal and nordamnacanthal were originally isolated from *Damnacanthus major* [25]. Previous studies on the synthesis of damnacanthal and nordmnacanthal were reported by Hirose, Roberts and Saha [26,27,28]. The current study describes the total synthesis of damnacanthal (**1**) and nordamnacanthal (**2**) with modified reaction steps, their cytotoxic activities of against MCF-7 and K-562 cancer cell lines and their structure activity relationships (SARs).

## 2. Results and Discussion

Anthraquinone skeletons are generally synthesized by Friedel-Crafts acylation condensation between phthalic anhydride and benzene derivatives [29]. 1,3-Dihydroxy-2-methylanthraquinone (**3**) was used as the common precursor for the synthesis of damnacanthal (**1**) and nordamnacanthal (**2**). This was synthesized by mixing of phthalic anhydride and 1,3-dihydroxy-2-methylbenzene in a molten mixture of AlCl_3_/NaCl [29,30]. 

**Figure 1:**
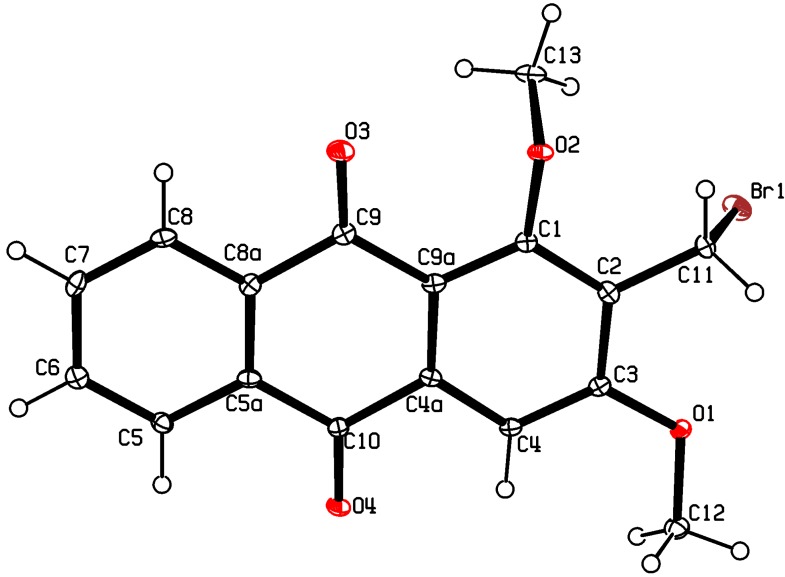
The ORTEP diagram of 2-bromomethyl-1,3-dimethoxy-9,10-athraquinone (**5**).

**Scheme 1:**
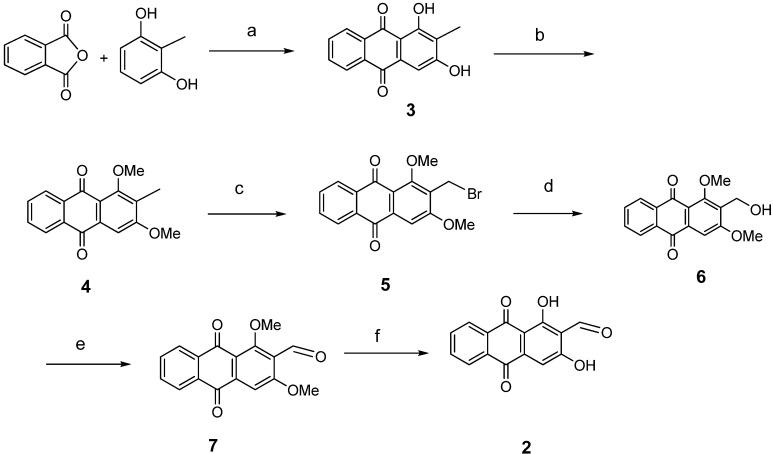
Reactions pathway for synthesis of nordamnacanthal (**2**).

The synthesis of nordamnacanthal (**2**) was accomplished by first acetylating the precursor compound **3** with acetic anhydride and potassium carbonate to afford the monoacetylated intermediate **8**. Upon methylation of compound **8** with K_2_CO_3_/(CH_3_)_2_SO_4_ in dry acetone to afforded 1,3-dimethoxy-2-methylathraquinone (**4**), which was then brominated with Wohl-Ziegler’s reagent (*N*-bromosuccinimide) in dry CCl_4_ to yield 2-bromomethyl-1,3-dimethoxyathraquinone (**5**) [25,31,32] and structure was confirmed by single X-ray diffraction (Figure 1). It is noteworthy that the use of a catalytic amount of benzoyl peroxide in this reaction gave 2-dibromomethyl-1,3-dimethoxyanthraquinone [26]. Compound **5** was hydrolyzed by refluxing it in acetic acid-water (8:2) to give the desired 2-hydroxymethyl-1,3-dimethoxyanthraqunone (**6**) in quantitative yield [26,31]. Compound **6**, which contains a hydroxymethyl moiety, was converted into the corresponding aldehyde **7** in 92.3% yield using a mild oxidizing agent [pyridinium chlorochromate (PCC) in dry CH_2_Cl_2_ at 20–25 °C]. The use of an excess amount (1.5 equiv.) of PCC also gave the undesired 1-hydoxy-3-methoxyanthraquinone-2-carboxylic acid. Upon treating compound **7** with AlCl_3_/CH_2_Cl_2_, nordamnacanthal (**2**) was obtained in 28% yield. The detailed reaction conditions are shown in Scheme 1.

**Scheme 2:**
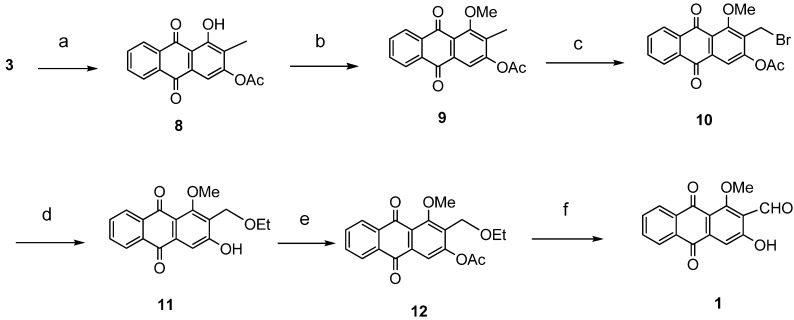
Reactions pathway for synthesis of damnacanthal (**1**).

The synthesis of damnacanthal (**1**) was accomplished by first acetylated the precursor compound **3** with (CH_3_)_2_SO_4_ and potassium carbonate to afforded monoacetylated derivative **8**. Upon methylation of compound **8** with K_2_CO_3_/CH_3_I compound **9** was obtained, which then brominated with NBS to yield 1-methoxy-3-aectoxy-2-bromomethyl-1-methoxyathraquinone (**10**) [25,32]. The bromo derivative **10** was converted into ethoxymethyl derivative **11** by dissolving it in a mixture of aq. NaOH/ethanol and followed by reflux in acidic media. Compound **11** was acetylated using acetic anhydride and K_2_CO_3_ to afforded **12**, which on oxidation with PCC in CH_2_Cl_2_ to give damnacanthal (**1**) in good yield. Thus the synthesis of damnacanthal (**1**) was achieved through modified steps as shown in Scheme 2.

*Reagents and conditions*: (a) anhydrous AlCl_3_, NaCl, 165–175 °C, 1 h; (b) Anhydrous K_2_CO_3_ (CH_3_)_2_SO_4_, dry acetone, refluxed, 22 h; (c) NBS/CCl_4_ refluxed for 30 h; (d) 80% acetic acid refluxed 24 h; (e) PCC, CH_2_Cl_2_ stir. 2–4 h. rt; (f) AlCl_3_ /CH_2_Cl_2_, HCl.

*Reagents and condition*s: (a) anhydrous K_2_CO_3_ /Ac_2_O, acetone; (b) anhydrous K_2_CO_3_/(CH_3_)_2_SO_4_ acetone, refluxed 22 h; (c) NBS/CCl_4_ refluxed 30 h; (d) 10% aq. NaOH/EtOH, aq. HCl; (e) Ac_2_O/ K_2_CO_3_ stirred for 24 h; (f) PCC, CH_2_Cl_2_ stir 2–4 h. rt.

**Figure 2:**
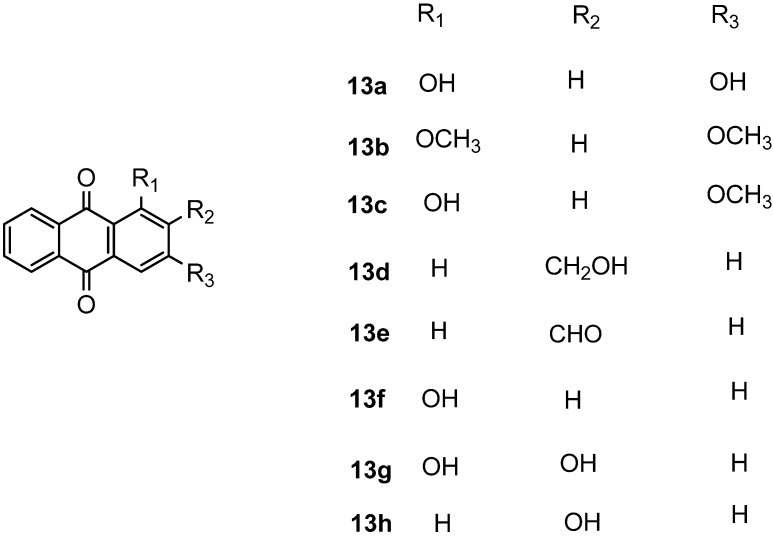
Structure of compounds **13a** to **13h**.

For the SARs additional anthraquinone analogous **13a**–**h** were synthesized by Friedel-Crafts acylation of phthalic anhydride with resorcinol or catechol to yielded respective anthraquinones [29,30] as shown in Figure 2. All compounds showed significant *in vitro* cytotoxicity against two cancer cell lines, indicating that anthraquinone is an interesting class of compounds for cancer therapy. The compound damnacanthal (**1**) contains methoxy, formyl and hydroxyl groups at the 1, 2 and 3 positions that might be important for the cytotoxicity since the standard drug doxorubicin [33] also possesses an anthraquinone moiety. However, nordamnacanthal (**2**), **3**, **8**, **13a**, **13c**, **13f** and **13h** exhibited less cytotoxic effects on both the MCF-7 and K-562 cell lines, suggesting that a protected OH group at position 1 and 3 increase the solubility and might make it easy for the compounds to diffuse across the cellular membrane as investigated by a theoretical study of 188 drug-like compounds by MI-QSAR analysis [34,35], while for poorly soluble drugs dissolution could be the rate limiting step in the absorption process. The methylated compound **13b** (IC_50_ = 24.25 ± 2.46 μM and 22.01 ± 3.54 μM) showed the stronger cytotoxicity than **13a ** (IC_50_ = 82.08 ± 1.46 and 60.42 ± 5.33 μM) and **13c** (IC_50_ = 87.80 ± 2.01 and 64.96 ± 1.57μM). Other derivatives **13e** (IC_50_ = 43.64 ± 2.12 and 46.61 ± 12.37) and 2-hydroxymethylanthraquinone (**13d**) (IC_50_ = 49.58 ± 12.61and 50.84±14.12) showed poor cytotoxicity as shown in Table 1. However, previously synthesized compounds **13f**–**h** with mono or dihydroxyl groups at the 1 and 2 positions exhibited moderate cytotoxic activity [36]. Further derivatives as exemplified by compounds **13c**–**h** indicated that this series of compounds only exhibited moderate activities not exceeding that of damnacanthal (**1**), suggesting that functional groups such as formyl at C-2, methoxy at C-1, hydroxyl groups at C-3 (damnacanthal) and bromomethyl at C-2 are important pharmacophores for the anticancer activity. However, the active compound **5** ((IC_50_ = 15.83 ± 0.58 and 23.61 ± 3.28 μM) might involve another mechanism like perfusion. The overall cytotoxic compounds (**1**, ** 5**, **13b**) showed more selectivity towards cancer cell lines because of their side chains and might fulfill the Lipinski rule-of-5 and drug-like properties.

**Table 1:** IC_50_ values of compounds **1** to **13h** against MCF-7 and K-562 cell lines.

Compound No.	IC50 value (μM)		
	Chemical Structure	MCF-7	K562
**1**	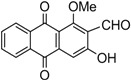	13.48 ± 2.02	19.50 ± 4.47
**2**	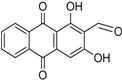	60.07 ± 6.46	32.46 ± 8.73
**3**	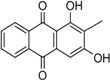	90.78 ± 1.49	100.71 ± 2.80
**4**	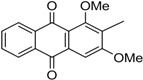	37.01 ± 13.82	87.81 ± 9.17
**5**	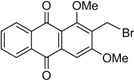	15.83 ± 0.58	23.61 ± 3.28
**6**	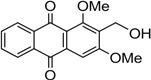	43.54 ± 0.48	47.62 ± 7.24
**7**	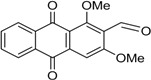	43.96 ± 3.42	49.66 ± 2.48
**8**	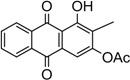	28.38 ± 7.94	69.59 ± 1.62
**9**	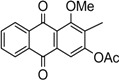	59.35 ± 8.23	46.77 ± 3.81
**10**	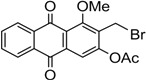	39.69 ± 6.24	54.90 ± 7.11
**11**	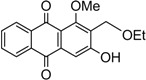	59.13 ± 11.59	42.06 ± 6.39
**12**	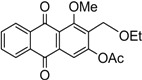	43.62 ± 5.35	89.36 ± 3.83
	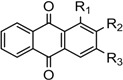 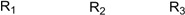		
**13a**	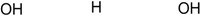	82.08 ± 1.46	60.42 ± 5.33
**13b**	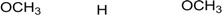	24.25 ± 2.46	22.01 ± 3.54
**13c**	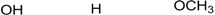	87.80 ± 2.01	64.96 ± 1.57
**13d**	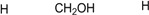	49.58 ± 12.61	50.84 ± 14.12
**13e**	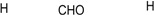	43.64 ± 2.12	46.61 ± 12.37
**13f**	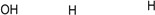	45.98 ± 2.95	107.14 ± 0.54
**13g**	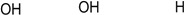	63.84 ± 12.46	70.54 ± 1.56
**13h**	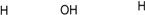	52.50 ± 2.63	94.17 ± 1.88
**Doxorubicin**	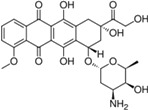	0.94 ± 0.20	0.24 ± 0.07

## 3. Experimental

### 3.1. General

Melting points were determined on a XSP-12 500X hot-stage melting point apparatus, and are uncorrected. UV spectra were recorded on a CARY 100 Conc. UV-visible spectrophotometer in MeOH. IR spectra were recorded on a Perkin–Elmer RXI Fourier Transform Infrared spectrometer (FTIR) as KBr discs. Mass spectra were measured on a Finnigan Mat SSQ 710 Spectrometer with ionization induced by electron impact at 70 eV. NMR spectra were recorded in CDCl_3_ and acetone-*d*_6_ using a Varian 500 MHz NMR spectrometer. Column chromatography was performed on silica gel (60 Merck 9385, 230–400 mesh, ASTM). The molecular structure of the intermediate 1,3-dimethoxy-2-bromomethylanthraquinone (**5**) was determined by X-ray crystallography. The data were collected at 100K using a Bruker APEXII with a CCD area-detector X-ray diffractometer. The structure was solved by direct method with SHELXS97 program and refined on F2 by full-matrix least-squares methods with anisotropic non-hydrogen atoms. The compound crystallizes in the monoclinic P2(1)/c space group with the crystallographic detail given in Table 2. The molecule exists as a planar molecule with r.m.s deviation of 0.018Å (Figure 1).

**Table 2:** Crystal data and structure refinement for 2-bromomethyl-1,3-dimethoxyanthraquinone (**5**).

Empirical formula	C_17_H_13_O_4_Br
Formula weight	361.18
Temperature	100(2) K
Wavelength	0.71073 Å
Crystal system	Monoclinic
Space group	*P2(1)/c*
Unit cell dimensions	*a* = 4.7401(8)Å, α = 90°
	*b* = 17.364(3)Å, β = 94.543(2)°
	*c* = 16.825(3)Å, γ = 90°
Volume	1380.5(4) Å^3^
Z	4
Calculated density	1.738 Mgm^−3^
Absorption coefficient	2.994 mm^−1^
F(000)	728
Crystal size	0.20 × 0.10 × 0.10 mm
θ range for data collection	1.69–28.26°
Limiting indices	−6 ≤ h ≤6, −14 ≤ k ≤23, −22 ≤ l ≤ 16
Reflections collected	3404
Independent reflections	2522 [R (int) = 0.0494]
Completeness to θ = 26.00º	99.40%
Absorption correction	Multi-scan
Max. and min. transmission	0.5973 and 0.7457
Refinement method	Full-matrix least-squares on F^2^
Data / restraints / parameters	3404/0/201
Goodness-of-fit on F^2^	1.038
Final R indices [I>2σ(I)]	R1 = 0.0417, wR2 = 0.0984
R indices (all data)	R1 = 0.0633, wR2 = 0.1124
Largest difference peak and hole	0.521 and −0.782 e.Å^−3^

### 3.2. General Procedure for Anthraquinone Synthesis

A mixture of anhydrous aluminium chloride (30 g, 225.0 mmol) and sodium chloride (12 g, 205.0 mmole) were melted at 125–130 °C. Phthalic anhydride (6.7 g, 45.0 mmole) and 2-methyl resorcinol (40.5 mmole) were mixed well and added slowly into the molten mixture of aluminum chloride and sodium chloride. The reaction temperature was raised till to 165–175 °C and maintained for 45–60 min. Another portion of anhydrous aluminum chloride (30 g, 225.0 mmole) was added into the hot mixture of the reactants [28]. After being cooled, the deep red solid product was decomposed by adding a mixture of ice water (250 mL) and conc. hydrochloric acid (250 mL). The crude mixture was dissolved in distilled water and organic layer was extracted with ethyl acetate, washed with brine and dried over anhydrous sodium sulfate. The crude products were purified by flash silica gel column chromatography with elution of the ethyl acetate /hexane as yellow needles.

*1,3-Dihydroxy-2-methylanthraquinone* (**3**). Yellow needles: 65% yield: m.p. 280–282 °C; {lit. [27] 280–283 °C}. UV (nm) in MeOH: 408, 279, 251. IR (cm^−1^): 3402 (OH), 2930, (C-H), 1661 (C=O non-chelated), 1628, 1591 (C=C< aromatic), 1338, 1310, 1122, 712. ^1^H-NMR (acetone-d_6_): δ 13.22 (s, 1H, 1-OH), 8.13 (dd, 1H, *J* = 1.5, 7.5 Hz, H-8), 8.23 (dd, 1H, *J* = 1.5, 7.5 Hz, H-5), 7.95–7.90 (m, 2H, H-6 & H-7), 7.37 (1H, s, H-4), 2.19 (3H, s, CH_3_). MS *m/z* (rel.int.): 254 (M^+^, 100), 226, 152 (10), 128 (21), 105 (11).

*2-Bromomethyl-1,3-dimethoxyanthraqinone* (**5**). To a solution of compound **4** (400 mg. 1.4 mmole) in dry CCl_4_ (40 mL), *N*-bromosuccinimide (500 mg, 3.0 mmole) was added portionwise. The whole mixture was stirred for 30 h. Compound **5** was extracted with ethyl acetate, filtered and purified by passing through a short column containing anhydrous sodium sulfate. The compound was recrystallized in a mixture of EtOAc and drops of MeOH as yellow crystals. Yield 90%; m.p.: 154–155 °C; {lit [27] 159–160 °C}. IR (cm^−1^): 2938 (CH), 1670 (C=O non-chelated), 1589 (C=C< aromatic), 1330, 1288, 1231, 1161, 730. ^1^H-NMR (CDCl_3_): δ 8.30 (d, 1H, *J* = 8.0 H_Z_, H-8), 8.25 (d, 1H, *J* = 8.0 Hz, H-5), 7.80–7.75 (m, 2H, H-6 & H-7), 7.26 (s, 1H, H-4), 4.76 (s, 2H, -CH_2_Br), 4.05 (s, 3H, OCH_3_), 3.97 (s, 3H, OCH_3_). MS *m/z* (rel.int.): 361 (M^+^+H, 38) 362 (M^+^+2, 37), 281 (100), 267 (35), 253 (36), 236 (42), 221 (7), 105 (11), 223 (41), 77 (21).

*1,3-Dihydroxy-2-formylanthraquinone (nordamnacanthal)* (**2**). Compound **7** (150 mg, 0.5 mmole) was suspended in dry CH_2_Cl_2_ (10 mL), stirred for 10 min. at rt and anhydrous AlCl_3_ (500 mg, 3.7 mmole) was added and stirred for 1h and then reflux for 4–6 h. The reaction mixture was cooled and added mixture of ice HCl (250 mL) and water (250 mL). The products was extracted by EtOAc and purified by CC and followed by recrystallized from a mixture of MeOH/EtOAc as orange crystals. Yield 28%; m.p.: 217–218 °C; {lit. [25] 220–221 °C}. UV (nm) in CHCl_3_: 422, 292, 263, 250. IR (cm^−1^): 3436 (OH), 2928, 2927, (CH), 1630 (C=O), 1560 (>C=C< aromatic), 1331, 1108, 786, 715. ^1^H-NMR (MeOD): δ 10.34 (s, 1H, CHO), 8.29 (d, 1H, *J* = 7.0 Hz, H-8), 8.25 (d, 1H, *J* = 7.0 Hz, H-5), 7.85–7.82 (m, 2H, H-6 & H-7), 7.29 (s, 1H, H-4). MS *m/z* (rel.int.): 268 (M^+^, 65) (10), 281 (100), 249 (100), 212 (34), 184 (32), 1197(24), 155 (7), 138 (12), 128 (21), 77 (12).

*2-Formyl-3-hydroxy-1-methoxyanthraquinone* (**1**). Compound **12** (200 mg 0.56 mmole) was dissolved in dry dichloromethane (30 mL) and PCC (125 × 2 mg, 1.39 mmole) was introduced slowly in a stirred solution. The reaction mixture was stirred at room temperature for 4–6 h. A black brown solid was obtained, dissolved in aqueous solution and extracted with EtOAc, solvent removed *in vacuo* on a rotary evaporator and dried over anhydrous sodium sulfate. The product was purified by column chromatography using EtOAc/hexane as solvent system. The product was obtained as orange crystals. Yield 64.5%; m.p.: 209–211 °C; (lit. (25) 211–212 °C. IR (cm^−1^): 3434 (OH), 2957, 2927, (CH), 1670 (non-chelated C=O), 1648 (C=O non-chelated), 1566 (C=C< aromatic), 1344, 1260, 1231, 1132, 7310. UV (nm) in CHCl_3_: 412, 284, 264, 254. ^1^H-NMR (acetone-d_6_): δ 10.45 (s , 1H, CHO), 8.32 (dd, 1H, *J* = 1.5, 7.5 Hz, H-8), 8.26 (dd, 1H, *J* = 1.5, 7.5 Hz, H-5), 7.87–7.78 (m, 2H, H-6 & H-7) 7.69 (s, 1H, H-4), 4.11 (s, 3H, OCH_3_). MS *m/z* (rel.int.): 282 (M^+^, 100) 267 (35), 254 (100), 237 (16), 225 (7), 197 (24), 180 (31), 168 (6) 152 (9), 139 (41).

### 3.3. X-ray Crystallography of Compound 5

A single crystal of compound **5** was obtained by slow evaporation at room temperature from a mixture of ethyl acetate-MeOH (drops). The crystal structure was solved by direct method with SHELXS97 programe and refined on F^2^ by full-matrix least-squares methods with anisotropic non-hydrogen bond atoms. The compound crystallized as a monoclinic *P2(1)/c* space group with the crystallographic data is given in Table 2. The molecule exists as a planar molecule with r.m.s deviation of 0.018 Å (Figure 1). There is no evidence of any hydrogen bonding interaction or π–π stacking present in the crystal packing of the compound. Full crystallographic data for structures **5** has been deposited at the Cambridge Crystallographic Data Center (CCDC) as supplementary publication number CCDC870566. Copies of the data can be obtained, free of charge, on application to CCDC 12 Union Road, Cambridge CB2 1EZ, UK [Fax: þ44 1223 336033. email: deposit@ccdc.cam.ac.uk or at www.ccdc.cam.ac.uk].

## 4. Cytotoxicity Assay

### 4.1. Preparation of Compounds

The compounds were dissolved in dimethylsulfoxide (DMSO, Sigma, St. Louis., MO, USA) to get a stock solution of 1 mg/mL and stored at 4 °C.

### 4.2. Preparation of Cell Lines

Adherent human estrogen dependent breast carcinoma MCF-7 and suspension human chronic myelogenic leukemia K-562 cell lines were obtained from ATCC, USA. Both MCF-7 and K562 were cultured in RPMI-1640 supplemented with 10% FBS and incubated at 37 °C, 5% CO_2_ and 90% humidity throughout the study. Cell viability was assessed by trypan blue exclusion method where both MCF-7 and K562 cell lines were collected, washed with PBS and mixed with 1:1 ratio of trypan blue dye and the cell number and viability were evaluated using haemacytometer under inverted light microscope. Only cell with viability higher than 95% will be subjected to MTT cell viability assay.

### 4.3. Cell Viability Assay

The effect of all compounds on cell viability of MCF-7 and K-562 cell lines were determined using the colorimetric 3-(4,5-dimethylthiazol-2-yl)-2,5-diphenyltetrazolium bromide (MTT) assay [37]. MCF-7 cell were seeded on a 96 well microtiter plate with each well containing 200 µL of culture media with 10% of FBS per well at a density of 1 × 10^5^ cells/mL and incubated overnight at 37 °C, 5% CO_2_ at humidified atmosphere of 95% air. When cells were reached 70% confluence then subjected to the cell viability assay. Medium containing the seeded MCF-7 cell was discarded. Treatment of both cultures were carried out by first adding 100 µL of RPMI-1640 medium supplemented with 10% of FBS into all wells except row A of the 96 well plates (TPP, Zollstrasse, Trasadingen, Switzerland). Then, 100 µL of each diluted compound (60 µg/mL) was added into row A and row B. A series of two-fold dilutions of compounds were carried out from row B to row G to give concentrations of 30, 15, 7.5, 3.75, 1.375 and 0.687 µg/mL, while row H was left as untreated control cell. For K-562 culture plate, 100 µL of cells (2 × 10^5^ cells/mL) was added into all wells. For MCF-7 culture plate, 100 µL of RPMI-1640 medium was added into all wells. The plates were incubated in 37 °C, 5% CO_2_ and 90% humidity incubator for 72 h. After incubation period, 20 µL of MTT (Sigma, 5 mg/mL) was added into all wells and the plates were further incubated for 4 h. Then, 170 µL of supernatant was aspirated from every well plate. The plates containing K-562 culture were centrifuged at 200 × g for 5 min prior to medium aspiration. The resulting formazan crystals in each well were solubilized by 100 µL of DMSO (Fisher Scientific, Waltham, MA, USA) followed by incubation for 20 min. Finally, the plates were read at 570 nm and 630 nm as reference wavelength by using µQuant ELISA Reader (Bio-tech Instruments, Winooski, VT, USA). The results of the compound-treated cells were compared with the standard doxorubicin. Each compound and control was assayed in triplicates in three independent experiments. The percentage of inhibitions were calculated by using Graph pad and expressed in µg/mL or µM and listed in Table 1. 

## 5. Conclusions

The synthesis of damnacanthal (**1**) and nordamnacanthal (**2**) was achieved. The key difference from the previously synthesis was the conversion of hydroxymethylanthraquinones **7** or **12** into the corresponding aldehyde, which was achieved by the use of optimized mild conditions with PCC as oxidant. The synthesis in good yield of 2-ethoxymethyl-3-hydroxy-1-methoxyanthraquinone (**11**) was achieved through a short sequence. The structure activity relationship suggested that methoxy, formyl, bromomethyl and hydroxyl groups are important for the cytotoxicity and selectivity in these substituted anthraquinones. These side chain modifications give an idea for further efforts to increase the therapeutic potential of this class of compounds. 
